# Application of Transesophageal Echocardiography in Amplatzer Atrial Septal Defect Occluder for Percutaneous Closure of Large Patent Foramen Ovale

**DOI:** 10.1155/2022/3226080

**Published:** 2022-07-22

**Authors:** Yajuan Du, Hang Xie, Hui Shao, Gesheng Cheng, Lu He, Xingye Wang, Xumei He, Beidi Lan, Yushun Zhang, Gang Tian

**Affiliations:** ^1^Department of Structural Heart Disease, The First Affiliated Hospital of Xi'an Jiaotong University, Xi'an, Shaanxi Province, China; ^2^Department of Cardiology, The First Affiliated Hospital of Xi'an Jiaotong University, Xi'an, Shaanxi Province, China

## Abstract

**Objective:**

The Amplatzer patent foramen ovale (PFO) occluder is the most commonly used device for percutaneous closure of a large PFO. However, its use may predispose the patient to postoperative residual shunting. To reduce the incidence of residual shunting, we investigated the safety and effectiveness of the Amplatzer atrial septal defect (ASD) occluder for percutaneous closure of a large PFO measured by transesophageal echocardiography (TEE) and evaluated the value of TEE in this procedure.

**Methods:**

Overall, 118 patients who were diagnosed with a large PFO (all with *a* ≥ 2 mm left atrial side height after the Valsalva maneuver (VM) excluding those with a small ASD) using contrast transthoracic echocardiography (c-TTE) and TEE underwent closure under TEE guidance at The First Affiliated Hospital of Xi'an Jiaotong University. An ASD device was used in 48 patients (group I) and a PFO device in 70 (group II). After the procedure, we verified the safety and efficacy of different devices using c-TTE, TTE, and TEE.

**Results:**

In both groups, the preoperative TEE results showed a significantly increased left height of the PFO after VM compared with that at rest (all *P* < 0.01). Compared with the left height of the PFO measured using TEE after VM, the PFO-stretch diameter (SD) measured by TEE after the delivery sheath passed the PFO was higher (all *P* < 0.01). We selected the ASD occluder size according to this PFO-SD. In group II, most patients underwent the implantation of the larger PFO devices. Interventional treatment was successfully performed on all patients. The effective occlusion rate in group I at 12 months after the procedure was significantly higher than that in group II (93.7% vs. 78.6%, *P* < 0.05). The TEE results showed that 18 patients with a medium and large residual shunt at 12 months after the procedure exhibited an intradisc tunnel-like shunt.

**Conclusion:**

The Amplatzer ASD device and Amplatzer PFO device are safe for large PFO closure, but the Amplatzer ASD device has a higher effective occlusion rate. TEE plays a crucial role in the use of the Amplatzer ASD occluder for percutaneous closure of a large PFO.

## 1. Introduction

Patent foramen ovale (PFO) is reported to occur in about 15%-25% of the general healthy adults [1, 2] and often results in a right-to-left shunt (RLS) when the right atrial pressure exceeds the left atrial pressure. Then, if a venous thrombus passes through a PFO, flows into the left side heart chamber, and reaches the arterial system, a paradoxical embolism develops [3]. PFO has been implicated in a number of clinical syndromes, including cryptogenic stroke, migraine, platypnea-orthodeoxia syndrome, and decompression sickness [4–7]. The hemodynamic basis for the emergence of these clinical symptoms is the RLS of PFO. Percutaneous PFO closure has been proven safe and effective for ensuring RLS abolition, and it can reduce the risk of recurrent cryptogenic stroke compared with medical therapy [8–11]. Residual shunt is the most common complication after PFO occlusion. Small residual shunts do not appear to have clinical significance [12]. However, patients with moderate-to-large residual right-to-left shunt (rRLS) have been shown to have a 4-fold increased risk of recurrent neuroembolic events [13]. A preoperative PFO with a large sized, long tunnel, small atrial septal defect (ASD) and improper selection of occluder device may be the main reasons for postoperative residual shunt. The most used device is Amplatzer PFO occluder 25 mm, but complex anatomy PFOs (such as large PFO and PFO with atrial septal aneurysm (ASA)) require larger device (Amplatzer PFO occluder 30 or 35 mm) for closure [14]. In previous studies, PFO with a balloon-stretched diameter larger than 11 mm or 13 mm was closed with a correspondingly sized Amplatzer ASD device [14, 15]. However, the left PFO height ≥2 mm after VM measured by transesophageal echocardiography (TEE) was defined as a large PFO [11, 16]. At present, no studies have observed the efficacy of an ASD occluder for closing a large PFO identified by TEE and the specific application of TEE in this procedure. Herein, we investigated the safety and efficacy of the Amplatzer ASD occluder for percutaneous closure of a large PFO measured by TEE and evaluated the value of TEE in this procedure.

## 2. Materials and Methods

### 2.1. Patient Population

From January 2019 to July 2020, 559 patients with PFO underwent closure at The First Affiliated Hospital of Xi'an Jiaotong University. Among them, 118 patients who were diagnosed with a large PFO (all with a ≥ 2 mm left atrial side height after VM, excluded PFO with small ASD) using contrast transthoracic echocardiography (c-TTE) and TEE were enrolled in this study. Among them, the following devices were used: Amplatzer ASD device in 48 patients (group I) and Amplatzer PFO device in 70 (group II). All patients were randomized to group I versus group II. They all provided written informed consent to participate in the study. The study protocol was approved by the ethics committee of The First Affiliated Hospital of Xi'an Jiaotong University. Routine ultrasound, computed tomography, and magnetic resonance imaging were used to rule out cardiac, intracranial, and extracranial arterial diseases and pulmonary arteriovenous malformations (PAVM).

### 2.2. TTE and TEE Examinations

**Figure 1:**
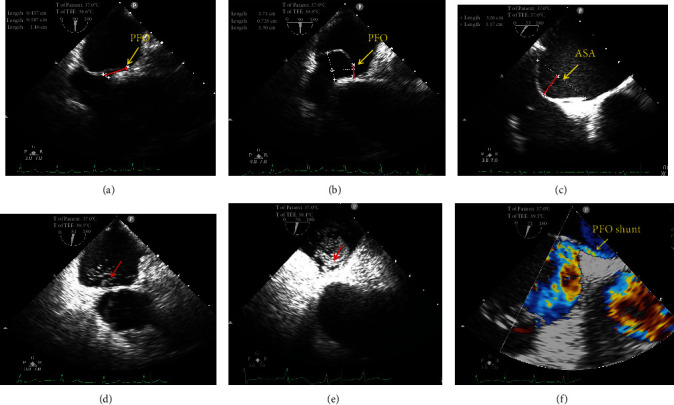
TEE detection of a PFO. (a) The left PFO height and PFO tunnel length were measured at rest on TEE (red lines). (b) The left PFO height after VM on TEE (red line). (c) The atrial septal excursion range on TEE (red line). (d) c-TEE showing a medium RSL through the PFO at rest. (e) c-TEE showing a large RLS through the PFO after VM. (f) Doppler color flow image showing a left-to-right shunt of the PFO. ASA: atrial septal aneurysm; c-TEE: contrast transesophageal echocardiography; PFO: patent foramen ovale; RLS: right-to-left shunt; TEE: transesophageal echocardiography; VM: Valsalva maneuver.

TTE and TEE were conducted using a GE Vivid E9 platform equipped with a 1.5-4.6 MHz M5S transducer and a 3.0-8.0 MHz multifrequency probe (Horten, Norway). The c-TTE was used for the initial PFO screening. The modified c-TTE procedure was performed according to the previously described methods [17]. The contrast TEE (c-TEE) was performed to confirm PFO presence. A standardized TEE protocol was used to respectively assess the PFO's anatomical and functional characteristics, such as its left height (at rest and after the VM), tunnel length, presence of an ASA, and presence of a hypermobile atrial septum (HAS), which were evaluated by an experienced sonographer [18] (Figure 1). PFO height after the VM was measured as the maximum separation between the septum primum and the septum secundum in the end-systolic frame (Figure 1(b)), and a height of ≥2 mm was defined as a large PFO [8]. An ASA was defined as a septal excursion ≥10 mm from the midline into the right or left atrium or ≥15 mm of the total excursion between the right and left atria. We also defined a floppy septum with a septal excursion ≥5 mm on every heartbeat as a HAS [19]. RLS was graded according to the highest number of microbubbles observed in the left chamber in a single frame as follows: image negative (no microbubbles), small (1-10 microbubbles), moderate (11-30 microbubbles), or large (>30 microbubbles or left chamber opacification) [20].

### 2.3. Occluder Device and Interventional Procedure

**Figure 2:**
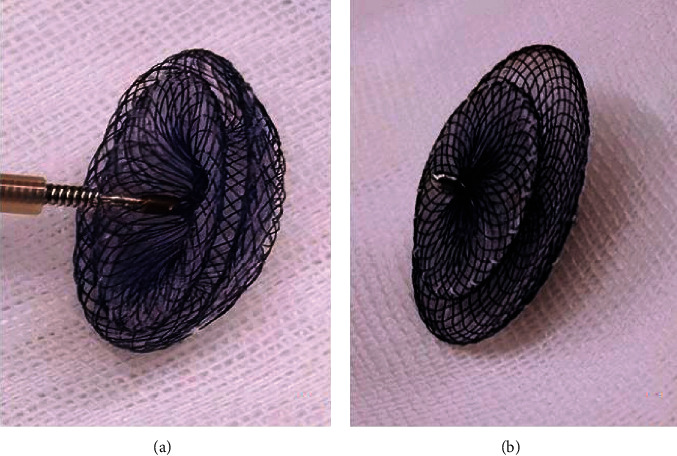
Amplatzer device. (a) The Amplatzer ASD device. (b) The Amplatzer PFO device. ASD: atrial septal defect; PFO: patent foramen ovale.

The 48 patients in group I were treated with the Amplatzer ASD devices, while the 70 patients in group II were treated with the Amplatzer PFO devices. Amplatzer ASD and PFO occluders are double-disc devices made from nitinol wires tightly woven into two flat discs with a connecting waist (Figure 2). The PFO occluder has smaller left disc and connecting waist and a larger right disc compared with the ASD occluder. The Amplatzer PFO occluder is presently available in 18/18, 18/25, 30/30, and 25/35 mm sizes for the left and right atrial disc size, respectively, while the Amplatzer ASD occluder is presently available in 22 sizes with a waist of 6-40 mm.

**Figure 3:**
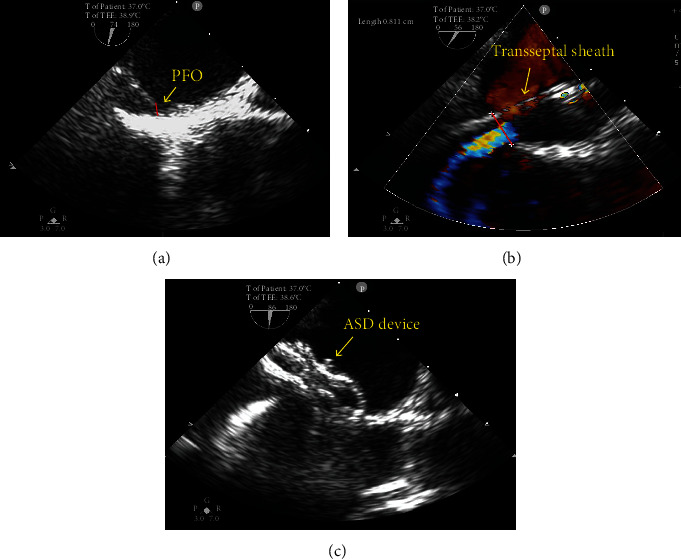
Implantation of the ASD device under TEE guidance. (a) The left height of the PFO after VM was 3 mm. (b) TEE displaying the 8 mm PFO-SD when the delivery sheath passed the PFO. (c) TEE displaying the proper shape and position of the 14 mm ASD device. ASD: atrial septal defect; SD: stretch diameter; PFO: patent foramen ovale; TEE: transesophageal echocardiography; VM: Valsalva maneuver.

All patients received aspirin 3-5 mg/kg/day and clopidogrel 75 mg/day up to 48 hours before the procedure, and all procedures were performed under general anesthesia under digital subtraction angiography and TEE guidance. The specific interventional procedures were performed as previously described [21]. The PFO-stretch diameter (SD) was measured using TEE after the delivery sheath passed the PFO in all patients. We selected the ASD device size according to the PFO-SD (Figure 3).

### 2.4. Management after Implantation

All patients continued taking aspirin 100 mg/day and clopidogrel 75 mg/day for 6 months, postoperatively. The second day after closure, patients underwent TTE to confirm correct occluder device position. TTE and c-TTE were performed at 6 and 12 months after closure. Eighteen patients with a medium or large residual shunt at 12 months after the procedure underwent TEE to reveal the reason for the residual shunt. At each visit, electrocardiography was performed.

### 2.5. Statistical Analysis

Continuous variables are expressed as mean ± standard deviations, while categorical variables are reported as counts and percentages. An unpaired two-tailed *t*-test was used to compare numerical variables, while the Chi-square or Fisher's exact test was used to compare categorical variables. The Wilcoxon rank-sum test was used to compare semiquantitative grading of the postoperative residual shunt in both groups. Statistical significance was assumed at *P* < 0.05. All data were analyzed using SPSS software (version 18.0.1, SPSS Inc.).

## 3. Results

### 3.1. Patients' Basic Information

There were no significant intergroup differences in the patients' baseline characteristics (Table 1). The preoperative TEE results showed that the left PFO height after VM was significantly increased compared with the height at rest (3.8 ± 0.2 mm vs. 1.6 ± 0.1 mm in group I and 3.5 ± 0.1 mm vs. 1.4 ± 0.1 mm in group II, *P* < 0.01). The PFO-SD measured TEE after the delivery sheath passed the PFO intraoperatively was significantly higher than the left PFO height after the VM (8.3 ± 0.4 mm vs. 3.8 ± 0.2 mm in group I and 8.2 ± 0.2 mm vs. 3.5 ± 0.1 mm in group II, *P* < 0.01).

### 3.2. Transcatheter Closure Results

We selected ASD device size according to the PFO-SD by TEE during the operation. The device size chosen was the measured PFO-SD plus 4-6 mm. In group I, the devices implanted were the following: two (4.2%) 10 mm ASD devices, three (6.3%) 12 mm ASD devices, 10 (20.8%) 14 mm ASD devices, seven (14.6%) 15 mm ASD devices, 13 (27.1%) 16 mm ASD devices, two (4.2%) 17 mm ASD devices, nine (18.8%) 18 mm ASD devices, and two (4.2%) 20 mm ASD devices. The average size of the selected ASD device was 15.5 ± 0.3 mm (Table 1). In group II, the larger Amplatzer PFO devices (30/30 and 25/35 mm) were implanted in 82.9% of the cases, and the devices implanted were the following: 12 (17.1%) 18/25 mm PFO devices, 37 (52.9%) 30/30 mm PFO devices, and 21 (30%) 25/35 mm PFO devices. The procedural success rate (without serious complications after hospitalization) was 100% in both groups.

### 3.3. Follow-Up Results

**Figure 4:**
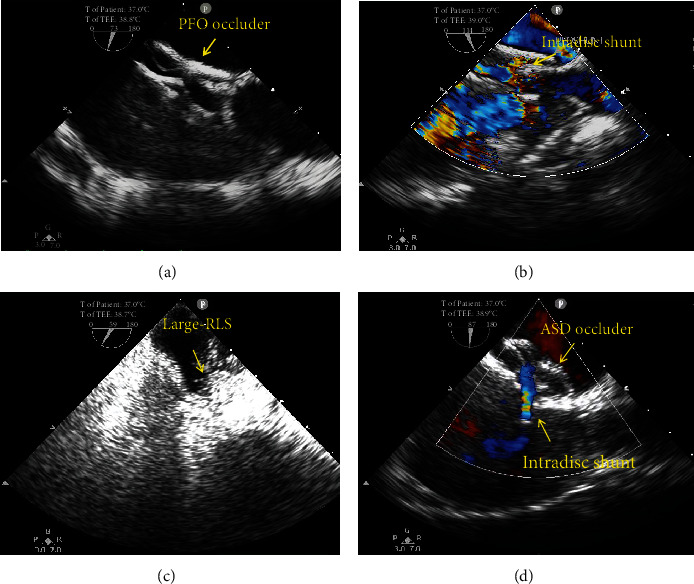
Two patients with a large residual RLS underwent TEE and c-TEE at 12 months after the procedure. (a) An oversized PFO occluder device. (b) An intradisc residual shunt. (c) A large residual RLS from the middle of the PFO occluder device. (d) An oversized ASD occluder device and an intradisc residual shunt. c-TEE: contrast transesophageal echocardiography; PFO: patent foramen ovale; RLS: right-to-left shunt; TEE, transesophageal echocardiography.

Follow-up was performed using TTE at 2 days after the procedure and using TTE and c-TTE at 6 and 12 months. All patients completed postoperative follow-up for the three periods. The TTE results showed the proper morphology and position of all devices in both groups. At 6 and 12 months after the procedure, the incidence of moderate and large residual shunts was significantly lower in group I than in group II (8.4% vs. 25.7% and 6.3% vs. 21.4%, all *P* < 0.05; Table 2). There were 19 patients with a small residual shunt in the two groups at 12 months after the procedure. According to the RLS characteristics of the c-TTE results, the 19 cases of a small residual shunt were considered physiological shunts between the pulmonary artery and vein. The effective occlusion rate at 12 months after the procedure was significantly higher in group I than in group II (93.7% vs. 78.6%, *P* < 0.05; Table 2). Among the 18 patients with a moderate and large residual shunt at 12 months after the procedure, 8 patients (only 1 in group I) had ASA and 3 patients (all in group II) had HAS. These 18 patients all underwent TEE. The result showed that the residual shunts were all intradisc (Figure 4). There was no evidence of novel arrhythmia, pericardial effusion, or other systemic responses in any patient.

## 4. Discussion

Many recent studies showed the efficacy of PFO closure for eliminating the RLS shunt and preventing recurrent stroke, particularly in patients with a large shunt [8–11, 22]. However, in clinical practice, residual shunts may be observed in up to 25% of patients after PFO closure, and nearly 10% show moderate to large residual shunts [8–11]. A study found that residual shunt, particularly of large size, is a novel risk factor independently associated with long-term stroke or transient ischemic attack recurrence [23]. A large preoperative PFO and improper selection of occluder device may be the prime reasons for a postoperative residual shunt. The larger Amplatzer PFO occluder is most commonly used for percutaneous closure of a large PFO. However, its use may predispose the patient to postoperative residual shunting. Wahl et al. aimed to assess the safety and clinical efficacy of PFO closure with Amplatzer PFO occluder under fluoroscopic guidance only. The results showed that patients with 18 and 25 mm devices (*n* = 542) had considerably fewer residual shunts compared with patients with 35 mm devices (*n* = 78), that is 7% versus 27%, respectively (*P* < 0.001). It indicated that larger devices, selected for larger shunts in the presence of an ASA, were associated with considerably higher residual shunt rates [24]. At present, no studies have observed the efficacy of an ASD occluder for closing a large PFO identified by TEE and the specific application of TEE in this procedure. Herein, to reduce the incidence of residual shunts, we aimed to explore the safety and efficacy of the Amplatzer ASD occluder for closing a large PFO measured by TEE and evaluate the value of TEE for this procedure.

The size and morphologic characteristics of PFO evaluated by TEE are important determinants of the clinical benefit of percutaneous device closure of PFO. In particular, for measuring PFO size, TEE has the irreplaceable advantage of TTE. The VM plays a crucial role in the TEE detection process. Previous studies have suggested the importance of VM in PFO diagnosis and specific measurements [25, 26]. After an effective VM, the right atrial pressure was higher than the left atrial pressure, while the PFO open diameter was significantly increased. As seen in our study, the preoperative TEE results showed a significantly increased left PFO height after VM compared to the height at rest in the two groups (3.8 ± 0.2 mm vs. 1.6 ± 0.1 mm in group I and 3.5 ± 0.1 mm vs. 1.4 ± 0.1 mm in group II, all *P* < 0.01). The importance of the VM in identifying a large PFO on TEE is also fully demonstrated. As shown in the previous studies, the left PFO height ≥2 mm after VM measured by TEE was defined as a large PFO [11, 16]. A large PFO often had a large RLS. In this study, all patients had a large shunt after the VM; RLS occurred at rest in 70.3% of the cases.

However, the PFO size measured by TEE after an effective VM is not its true size. The PFO's open diameter changes with the left and right atrial pressure; moreover, it changes more significantly under the action of mechanical support forces. To investigate the patient selection, efficacy, and safety of in-tunnel closure with a FlatStent EFTM, Noc et al. measured the maximal PFO diameter (6.3 ± 2.3 mm) by the stretched balloon during the procedure (preprocedural PFO diameter was 2.0 ± 0.5 mm on TEE) [27]. Giordano et al. reported that the maximal PFO diameter measured by the same method was used to select the Amplatzer ASD occluder device size and that the mean maximal PFO diameter was 14.91 ± 1.41 mm. The smallest balloon diameter needed to achieve a stop flow at echocardiographic evaluation addressed the choice ASD device size [14]. ASD device size selection was 2 mm larger than stop flow diameter [28]. In the present study, we measured the PFO-SD by TEE after the delivery sheath passed the PFO during the operation. The thick and hard delivery sheath through the atrial septum mechanically pulls open the soft primary septum and significantly increases the PFO's open diameter. Compared with the left PFO height measured after the VM by TEE, the PFO-SD measured using TEE during the operation was significantly higher in both groups (8.3 ± 0.4 mm vs. 3.8 ± 0.2 mm in group I and 8.2 ± 0.2 mm vs. 3.5 ± 0.1 mm in group II, *P* < 0.01). But despite this, the PFO-SD measured by TEE may still not be the maximal extension diameter of PFO. So, we attempted to consider a PFO-SD measured by TEE as a hard edge ASD and selected the size of the ASD occluder according to the principles reported in the previous literature: the ASD device size chosen is the measured (by TTE) ASD size plus 4-6 mm [29]. The average size of the selected ASD device was 15.5 ± 0.3 mm. In group II, the larger Amplatzer PFO devices (30/30 and 25/35 mm) were implanted in 82.9% of cases. The procedural success rate was 100% in both groups.

In our study, all patients underwent postoperative follow-up at 2 days, 6 months, and 12 months. There was no evidence of novel arrhythmia, pericardial effusion, or other systemic responses in any patients. The effective occlusion rate at 12 months after the procedure was significantly higher in group I than in group II (93.7% vs. 78.6%, *P* < 0.05; Table 2). Giordano et al. compared Amplatzer ASD occluder versus Amplatzer PFO occluder 30 or 35 mm about the safety of procedure and the presence of residual shunt during the follow-up. At the last follow-up, patients treated with ASD occluder and those treated with A-PFO 30/35 had complete closure percentage of 97% and 72.7% (*P* < 0.01), respectively [14]. This is similar to our result. It has been shown that the time of complete occlusion after implantation of a PFO occluder was more dependent on the anatomy of atrial septum than on the type of the device [30]. But in this study, there were no differences in anatomical characteristics of the preoperative PFO between group I and group II (Table 1). Based on this, we concluded that the Amplatzer ASD device has a higher effective occlusion rate for large PFO closure. The major effectiveness of Amplatzer ASD occluder could be due to the wide waist of the device that allows itself a higher capability to fill the large PFO. However, the size of ASD occluder we chose should not be too large. Eighteen patients with a moderate and large residual shunt at 12 months after the procedure underwent TEE. The results showed that the residual shunts were all intradisc. In group I, the reason for the moderate and large residual shunt in 3 cases may be the selection of a larger ASD device. Therefore, the two disks remain separated not adhering to the septum (Figure 4(d)). In group II, the residual shunt may be due to a larger PFO device being implanted in a larger PFO, poor fit of the occluder disc and atrial septum, and an overly thin occluder waist, which can easily cause the tunnel-like residual shunt in the occluder (Figure 4(a–c)). Among the 18 patients with a moderate and large residual shunt, 8 patients (only 1 in group I) had ASA and 3 patients (all in group II) had HAS. According to the report, the residual shunts were more prevalent in patients with an ASA and especially in those whom a 35 mm occluder was used [31]. They suggested that to overcome the problem of frequent residual shunts in patients with large ASA, the use of other devices, for example Amplatzer ASD occluder, might be considered [31]. That is similar to our view. In some patients with moderate and large residual shunts on bubble studies, other anatomic lesions (such as small ASDs or PAVMs) may coexist [32]. In this study, we excluded these lesions preoperatively. There were 19 cases of a small residual shunt in the two groups at 12 months after the procedure. According to the RLS characteristics of the c-TTE results, the small residual shunt in these 19 cases was considered a physiological shunt between the pulmonary artery and vein. The previous literature reports have shown that transpulmonary passage of small and moderate contrast bubbles may be traveling through larger diameter vessels. There is direct evidence that these larger diameter (>25-50 *μ*m) intrapulmonary arteriovenous anastomoses exist in healthy human, baboon, and dog lungs [33]. The intrapulmonary arteriovenous vessels that allow for the transpulmonary passage of saline contrast bubbles during normoxic and hypoxic exercise in adult humans may be remnant fetal pathways [34]. Different from PAVM, such anastomoses are located in the lung apices, closed at rest and in the upright position, but can be reopened in the supine position, by breathing a hypoxic air mixture or after strenuous exercise [35]. In our study, one year after occlusion, the small shunts detected by c-TTE may be originating from these physiological intrapulmonary arteriovenous anastomoses.

Our study has important clinical implications. The results indicated that the Amplatzer ASD occluder for closing a large PFO can reduce the incidence of moderate and large residual shunts and then maybe reduce the incidence of long-term stroke or transient ischemic attack recurrence. Furthermore, we selected Amplatzer ASD devices using the PFO-SD measured on intraoperative TEE instead of using the maximal PFO diameter by the stretched balloon, and that reduced the cost of the operation.

However, our study has some limitations. For instance, not all patients underwent a TEE examination at 6 and 12 months postoperatively, which made it impossible to determine the presence of an occluder-related thrombosis. Moreover, because of the small number of patients in our study, the real residual shunt rate requires further clinical experiments with large multicenter samples to verify the results. Moreover, the follow-up time was short; therefore, the long-term effects require further observations.

## 5. Conclusion

The present study preliminarily demonstrated the feasibility, safety, and efficacy of Amplatzer ASD devices for the closure of a large PFO measured by TEE. Neither device- nor procedure-related major adverse events occurred, and the PFO effective closure rate was 93.7%. TEE has a major role in the preoperative evaluation of PFO size and anatomy, the measurement of PFO-SD after the delivery sheath passed the PFO during the operation, and the assessment of postoperative residual shunt. We also preliminarily confirmed the feasibility of selecting Amplatzer ASD devices using the PFO-SD measured on intraoperative TEE.

## Figures and Tables

**Table 1:** Baseline clinical and anatomical data of the study population.

Baseline characteristics	Group I	Group II	*P*
	(*n* = 48)	(*n* = 70)	
Clinical features			
Age (years)	46.7 ± 1.6	44.2 ± 1.5	0.277
Sex (male)	18 (37.5)	23 (32.9)	
Sex (female)	30 (62.5)	47 (67.1)	0.603
Coronary heart disease	1 (2.1)	2 (2.9)	0.739
Hypertension	3 (6.2)	4 (5.7)	0.783
Arrhythmia			
Atrial premature beat	1 (2.1)	1 (1.4)	0.645
Indication for closure			
Cryptogenic stroke	6 (12.5)	13 (18.6)	0.378
Transient ischemic attack	11 (22.9)	18 (25.7)	0.729
Migraine	24 (50)	29 (41.4)	0.358
Transient syncope	2 (4.2)	5 (7.1)	0.783
Preprocedural c-TTE and TEE data			
Positive at rest by c-TTE	38 (79.2)	45 (64.3)	0.082
Large RLS after VM by c-TTE	48 (100)	70 (100)	
Left PFO height at rest by TEE (mm)	1.6 ± 0.1	1.4 ± 0.1	0.06
Left PFO height after VM by TEE (mm)	3.8 ± 0.2^a^	3.5 ± 0.1^a^	0.145
PFO tunnel length by TEE (mm)	9.3 ± 0.3	8.5 ± 0.3	0.09
ASA presence by TEE	16 (33.3)	27 (38.6)	0.561
HAS presence by TEE	6 (12.5)	9 (12.9)	0.491
PFO-SD and occluder device selection			
PFO-SD by TEE during operation (mm)	8.3 ± 0.4^b^	8.2 ± 0.2^b^	0.876
ASD device waist size (mm)	15.5 ± 0.3		
PFO device size			
18/25		12 (17.1)	
30/30		37 (52.9)	
25/35		21 (30)	

Compared with the left height of PFO by TEE at rest, ^a^*P* < 0.01; compared with the left height of PFO after VM by TEE, ^b^*P* < 0.01. ASD: atrial septal defect; c-TTE: contrast transthoracic echocardiography; HAS: hypermobile atrial septum; SD: stretch diameter; PFO: patent foramen ovale; TEE: transesophageal echocardiography; VM: Valsalva maneuver.

**Table 2:** Residual shunt rate of the PFO after closure by group.

c-TTE results	Group I (*n* = 48)	Group II (*n* = 70)	*P*
6-month follow-up			0.033
Negative	33 (68.8)	37 (52.9)	
Small residual RLS	11 (22.9)	15 (21.4)	
Moderate residual RLS	2 (4.2)	5 (7.1)	
Large residual RLS	2 (4.2)	13 (18.6)	
12-month follow-up			0.035
Negative	38 (79.1)	45 (64.3)	
Small residual RLS	7 (14.6)	12 (17.1)	
Moderate residual RLS	1 (2.1)	4 (5.7)	
Large residual RLS	2 (4.2)	11 (15.7)	

PFO: patent foramen ovale; RLS: right-to-left shunt.

## Data Availability

The raw data supporting the conclusions of this article will be made available by the corresponding author under a reasonable request.
